# Backdoors for Linear Temporal Logic

**DOI:** 10.1007/s00453-018-0515-5

**Published:** 2018-09-18

**Authors:** Arne Meier, Sebastian Ordyniak, M. S. Ramanujan, Irena Schindler

**Affiliations:** 1 Institut für Theoretische Informatik, Leibniz Universität Hannover, Appelstrasse 4, 30167 Hannover, Germany; 2 Algorithms Group, University of Sheffield, Regent Court, 211 Portobello, Sheffield, S1 4DP UK; 3 Institut für Computergraphik und Algorithmen 186/1, Technische Universität Wien, Favoritenstraße 9–11, 1040 Wien, Austria

**Keywords:** Linear temporal logic, Parameterized complexity, Backdoor sets

## Abstract

In the present paper, we introduce the backdoor set approach into the field of temporal logic for the global fragment of linear temporal logic. We study the parameterized complexity of the satisfiability problem parameterized by the size of the backdoor. We distinguish between backdoor detection and evaluation of backdoors into the fragments of Horn and Krom formulas. Here we classify the operator fragments of globally-operators for past/future/always, and the combination of them. Detection is shown to be fixed-parameter tractable whereas the complexity of evaluation behaves differently. We show that for Krom formulas the problem is paraNP-complete. For Horn formulas, the complexity is shown to be either fixed parameter tractable or paraNP-complete depending on the considered operator fragment.

## Introduction

Temporal logic is one of the most important formalism in the area of program verification and validation of specification consistency. Most notable are the seminal contributions of Kripke [21], Pnueli [32], Emerson, Clarke, and Halpern [7, 14] to name a few. There exist several different variants of temporal logic from which, best known are the computation tree logic CTL, the linear temporal logic LTL, and the full branching time logic CTL$^*$. In this paper, we will consider the global fragment of LTL for formulas in *separated normal form* (SNF) which has been introduced by Fisher [15]. This normal form is a generalization of the conjunctive normal form from propositional logic to linear temporal logic with future and past modalities interpreted over the flow of time, i.e., the frame of the integers $({\mathbb {Z}},<)$. In SNF the formulas are divided into a past, a present, and a future part. Technically this normal form is not a restriction since one can always translate an arbitrary LTL formula to a satisfiability-equivalent formula in SNF in time linear in the original formula [15]. In fact, the restriction to SNF normal form is crucial for us, because it is known that syntactical restrictions of arbitrary LTL formulas such as Horn or Krom do not lead to tractability [4].

LTL and its two main associated computational problems LTL model checking and LTL satisfiability have been deeply investigated in the past. In this work we focus on the LTL satisfiability problem, i.e., given an LTL formula the question is whether there is a temporal interpretation that satisfies the formula. Sistla and Clarke classified the computational complexity of the satisfiability problem to be $\mathsf {PSPACE}$-complete [36]. Then, later, several restrictions of the unrestricted problem have been considered. These approaches considered operator fragments [29], Horn formulas [4], temporal operator fragments, temporal depth, and number of propositional variables [8], the use of negation [27], an XOR fragment [11], an application of Post’s lattice [3], and the SNF fragment [2].

In contrast to LTL satisfiability where the search for fruitful parameterization has so far been rather unsuccessful [26], various important parameterizations have been identified for the satisfiability problem of propositional formulas (SAT) [5, 30, 37]. One very prominent and well-studied structural parameterization for SAT are so-called backdoor sets. Informally, backdoors are small sets of variables of a SAT instance that represent “clever reasoning shortcuts” through the search space. Backdoor sets have been widely used in the areas of propositional satisfiability [9, 10, 19, 20, 33, 35, 38], and also for material discovery [25], abductive reasoning [31], argumentation [13], planning [22, 23], and quantified Boolean formulas [34]. A backdoor set is defined with respect to some fixed *base class* for which the computational problem under consideration is polynomial-time tractable. For instance, in the case of SAT, a backdoor set *B* for a given CNF formula $\phi$ into the base class of Horn formulas is a set of variables such that for every assignment of the variables in *B* it holds that the reduced formula, i.e., the formula obtained after applying the assignment to $\phi$, is Horn. Given such a backdoor set one can decide the satisfiability of $\phi$ in time $O(2^{|B|}p(|\phi |))$ by enumerating the $2^{|B|}$ assignments of the variables in *B* and for each such assignment solving the remaining formula in time $p(|\phi |)$, where *p* is a polynomial given by the base class. As a result, once a small backdoor set is identified the satisfiability check is *fixed-parameter tractable* for the parameter backdoor size. Since the backdoor set is usually not provided with the input, it is crucial that small backdoor sets to a given base class can be found efficiently. When employing the backdoor approach one consequently usually considers two subtasks the so-called *detection* and *evaluation* problem, where the former is the task to identify a small backdoor set and the later concerns the solution of the problem using the backdoor set.

**Table 1:** Results overview

Problem	Operators	$\textsc {horn}$		$\textsc {krom}$	
Detection	Any	$\mathsf {FPT}$	(Thm. 5)	$\mathsf {FPT}$	(Thm. 5)
Evaluation		$\mathsf {FPT}$	(Thm. 8)	$\mathsf {paraNP}$-c.	(Thm. 9)
	${{\mathrm{\Box _{\mathrm {F}}}}},{{\mathrm{\Box _{\mathrm {P}}}}}$	$\mathsf {paraNP}$-c.	(Thm. 10)	$\mathsf {paraNP}$-c.	(Above)
	One of ${{\mathrm{\Box _{\mathrm {F}}}}},{{\mathrm{\Box _{\mathrm {P}}}}}$	Open		$\mathsf {paraNP}$-c.	(Cor. 11)
LTL-SAT		$\mathsf {P}$ [2]		$\mathsf {NP}$-c. [2]	
		$\mathsf {P}$ [2]		$\mathsf {NL}$ [2]	

The term “Any” refers to any combination of , whereas “Above” denotes that the lower bound from the cell above applies

*Our Contribution* In this paper, we introduce a notion of backdoors for the global fragment of  formulas that are given in SNF. Namely, we consider backdoor sets to the base classes that have recently been identified by Artale et al. [2]. These base classes are defined by both restrictions on the allowed temporal operators (i.e., to a subset of ) and restrictions on the clauses to be either $\textsc {horn}$ or $\textsc {krom}$. We show that surprisingly a notion of backdoor sets very similar to the strong backdoor sets employed for SAT [18] can also be successfully applied to  formulas. Whereas the detection of these backdoor sets can be achieved via efficient fpt-algorithms for all the considered fragments (using algorithms similar to the algorithms employed in the context of SAT), the evaluation of these backdoor sets turns out to be much more involved. In particular, we obtain tractability of the evaluation problem for $\textsc {horn}$ formulas using only the always operator. In fact,  restricted to only the always operator, is already quite interesting, since it allows one to express “Safety” properties of a system. For almost all of the remaining cases we show that the evaluation problem is $\mathsf {paraNP}{}$-hard. Moreover, the techniques used to show these results are very different from and more involved than the techniques employed for SAT, i.e., in the context of SAT the backdoor set evaluation problem is trivial. Our results are summarized in Table 1.

## Preliminaries

*Parameterized Complexity* A good introduction into the field of parameterized complexity is given by Downey and Fellows [12]. A *parameterized problem*$\varPi$ is a tuple $(Q,\kappa )$ such that the following holds. $Q\subseteq \varSigma ^*$ is a language over an alphabet $\varSigma$, and $\kappa :\varSigma ^*\rightarrow \mathbb N$ is a computable function; then $\kappa$ also is called the *parameterization (of*  $\varPi$).

If there is a deterministic Turing machine *M* and a computable function $f:\mathbb N\rightarrow \mathbb N$ s.t. for every instance $x \in \varSigma ^*$ (i) *M* decides correctly if $x \in Q$, and (ii) *M* has a runtime bounded by $f(\kappa (x)) \cdot |x|^{O(1)}$, then we say that *M* is an *fpt-algorithm for* $\varPi$ and that $\varPi$ is *fixed-parameter tractable* (or in the class $\mathsf {FPT}$). If *M* is non-deterministic, then $\varPi$ belongs to the class $\mathsf {paraNP}$. One way to show $\mathsf {paraNP}$-hardness of a parameterized problem $(Q,\kappa )$ is to show that *Q* is $\mathsf {NP}$-hard for a specific, fixed value of $\kappa$, i.e., there exists a constant $\ell \in \mathbb N$ such that $(Q, \kappa )_\ell \mathrel {\mathop :}=\{ x \mid x \in Q \text { and } \kappa (x) = \ell \}$ is $\mathsf {NP}$-hard.

*Temporal Logic* We assume familiarity with standard notions of propositional logic. Let $\mathsf {PROP}$ be a finite set of propositions and $\bot$/$\top$ abbreviate the constants false/true. The syntax of the global fragment of  is defined by the following EBNF:where $p\in \mathsf {PROP}$. Here ${{\mathrm{\Box _{\mathrm {P}}}}}\varphi$ can be read as “$\varphi$ holds in every point in the past”, ${{\mathrm{\Box _{\mathrm {F}}}}}\varphi$ as “$\varphi$ holds in every point in the future”, and  as “$\varphi$ holds always”. We also will make use of well-known shortcuts such as $\rightarrow ,\leftrightarrow$. Now we define the semantics of these formulas. Here, we interpret  formulas over the flow of time $(\mathbb Z,<)$ (for further information on this approach, see, e.g., Gabbay et al. [17]). Note that all our results can be easily transferred to the case if the formulas are evaluated over the set of natural numbers instead of the set of all integers.

### Definition 1

(*Temporal Semantics*) Let $\mathsf {PROP}$ be a finite set of propositions. A *temporal interpretation*$\mathfrak {M}=(\mathbb Z,<,V)$ is a mapping from propositions to moments of time, i.e., $V:\mathsf {PROP}\rightarrow {\mathcal {P}}(\mathbb Z)$. The satisfaction relation $\models$ is then defined as follows where $n\in \mathbb Z$, 
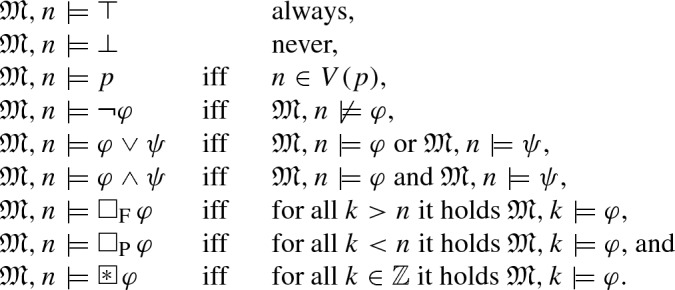


We say that $\varphi$ is *satisfiable* if there is a temporal interpretation $\mathfrak {M}$ such that $\mathfrak {M},0\models \varphi$. Then $\mathfrak {M}$ is also referred to as a *(temporal) model (of* $\varphi )$. Sometimes we also directly write $\mathfrak {M}(p)$ instead of *V*(*p*).

Table 2 exemplifies the semantics with some basic formulas. As shown by Fisher et al. every  formula considered over the frame $({\mathbb {Z}},<)$ has a satisfiability-equivalent formula in the separated normal form SNF [16], which can be constructed in linear time [15]). We follow the notation of SNF formulas by Artale et al. [2] and directly restrict them to the relevant global fragment of this study:12where $\lambda$ is called a *temporal literal* and $\varphi$ is said to be in *clausal normal form*.

Note that the operator name $\mathsf {G}$ instead of ${{\mathrm{\Box _{\mathrm {F}}}}}$ often occurs in literature. Yet, in contrast to $\mathsf {G}\varphi$, for ${{\mathrm{\Box _{\mathrm {F}}}}}\varphi$ it is not required that $\varphi$ holds in the present world. We distinguish fragments of  by adding superscripts and subscripts as follows. If  is an operator subset then  is the fragment of  consisting of formulas that are allowed to only use temporal operators from *O* for temporal literals, i.e., it is a constraint on the allowed operators in equation (1) from above. We also consider restrictions of the clausal normal form in (2): . Table 3 lists the relevant cases for this study. If $\alpha \in \{\textsc {cnf},\textsc {horn},\textsc {krom}\}$ then  is the set of formulas where the subformulas of the type , obey the normal form $\alpha$.

**Table 2:** Temporal semantics

	$<0$	0	1	2	3	4	5	$>5$
*p*	1	1	0	1	0	1	1	0
*q*	0	0	1	1	0	0	1	1
*r*	0	0	0	0	1	0	0	0
$p \wedge q$	0	0	0	1	0	0	1	0
	0	0	0	0	0	0	0	0
${{\mathrm{\Box _{\mathrm {F}}}}}q$	0	0	0	0	0	1	1	1
${{\mathrm{\Box _{\mathrm {F}}}}}{{\mathrm{\Box _{\mathrm {F}}}}}q$	0	0	0	0	1	1	1	1
${{\mathrm{\Box _{\mathrm {P}}}}}p$	1	1	1	0	0	0	0	0
	0	0	0	0	0	0	0	0
	1	1	1	1	1	1	1	1

**Table 3:** Considered normal forms

Class	Description	Restrictions on *n*, *m*
$\textsc {cnf}$	No restrictions on (2)	–
$\textsc {horn}$	At most one positive temporal literal	$m\le 1$
$\textsc {krom}$	Binary clauses	$n+m\le 2$

Restrictions refer to Eq. (2)

The following lemma shows a log-space constructible normal form which prohibits deep nesting of temporal operators of the investigated formulas.

### Proposition 2

([2, Lemma 2]) Let  be a formula class for $\alpha \in \{\textsc {cnf},\textsc {horn},\textsc {krom}\}$. For any formula $\varphi \in {\mathcal {L}}$, one can construct, in log-space, a satisfiability-equivalent ${\mathcal {L}}$-formula , where $\varPsi$ is a conjunction of propositional variables from $\varPhi$, and $\varPhi$ is a conjunction of clauses of the form (3) containing only ${{\mathrm{\Box _{\mathrm {F}}}}},{{\mathrm{\Box _{\mathrm {P}}}}}$ for , ${{\mathrm{\Box _{\mathrm {F}}}}}$ for , ${{\mathrm{\Box _{\mathrm {P}}}}}$ for , and only  for , in which the temporal operators are not nested.

In the following sections we consider only formulas given in this normal form .

## Introduction of Backdoors for the Global Fragment of LTL

In the following, we will introduce a notion of backdoors for formulas in the global fragment of linear temporal logic. The definition of these backdoors turns out to be very similar to the definition of the so-called strong backdoor sets for propositional formulas [18]. The main difference is that whenever a propositional variable is in the backdoor set then also all of its temporal literals are required to be in the backdoor set as well. A consequence of this is that in contrast to propositional formulas, where a backdoor set needs to consider all assignments of the backdoor set variables, we only need to consider assignments that are consistent between propositional variables and their temporal literals.

Let $\mathcal {O}$ be a set of operators. An assignment $\theta :\mathrm {Vars}(\phi )\cup \{\,Ox \mid x \in \mathrm {Vars}(\phi )\wedge O \in \mathcal {O}\,\}\rightarrow \{0,1\}$ is *consistent* if for every $x\in \mathrm {Vars}(\phi )$ it holds that if , then also $\theta (\Box _P x)=1$, $\theta (\Box _F x)=1$, and $\theta (x)=1$.

### Definition 3

(*Backdoors*) Let ${\mathcal {C}}$ be a class of $\textsc {cnf}$-formulas, ${\mathcal {O}}$ be a set of operators, and $\phi$ be an  formula. A set $X\subseteq \mathrm {Vars}(\phi )$ is a *(strong)*  $({\mathcal {C}},{\mathcal {O}})$-*backdoor* if for every consistent assignment $\theta :X\cup \{Ox\mid x\in X, O\in {\mathcal {O}}\}\rightarrow \{0,1\}$ it holds that $\phi [\theta ]$ is in ${\mathcal {C}}$.

The *reduct*$\phi [\theta ]$ is defined similarly to that for standard $\textsc {cnf}$-formulas, i.e., all clauses that contain a satisfied literal are deleted, and all falsified literals are deleted from their clauses. Here empty clauses are substituted by false, and the empty formula by true. Sometimes if the context of ${\mathcal {O}}$ is clear, we omit to state it and just mention the backdoor class ${\mathcal {C}}$.

### Example 4

Let  be the considered formula. Then $B=\{p_3\}$ is a strong -backdoor as the following assignments have to be examined:

**Table:** 

$p_3$	${{\mathrm{\Box _{\mathrm {P}}}}}p_3$		$\varphi [\theta ]$	
0	0	0		$\star$
0	0	1	Irrelevant as inconsistent	
0	1	0	$p_1\wedge p_2$	$\heartsuit$
0	1	1	Irrelevant as inconsistent	
1	0	0		$\star$
1	0	1	Irrelevant as inconsistent	
1	1	0	$p_1\wedge p_2$	$\heartsuit$
1	1	1	$p_1\wedge p_2$	

First, observe that all relevant rows lead to a $\textsc {krom}$-formula. Note that for the rows marked with $\star$ the reduct just removed the temporal literal ${{\mathrm{\Box _{\mathrm {P}}}}}p_3$. All other rows are either inconsistent (and hence irrelevant) or delete the clause $(\lnot {{\mathrm{\Box _{\mathrm {P}}}}}p_4 \vee {{\mathrm{\Box _{\mathrm {P}}}}}p_2\vee {{\mathrm{\Box _{\mathrm {P}}}}}p_3)$ completely, because ${{\mathrm{\Box _{\mathrm {P}}}}}p_3$ is set to true. At first glance, our definition of backdoor sets for LTL is almost purely syntactical, and thereby is an accordance to strong backdoor sets for the propositional satisfiability problem. For instance consider the assignments marked with the $\heartsuit$. In these cases we delete the clause $(\lnot {{\mathrm{\Box _{\mathrm {P}}}}}p_4 \vee {{\mathrm{\Box _{\mathrm {P}}}}}p_2\vee {{\mathrm{\Box _{\mathrm {P}}}}}p_3)$ completely because ${{\mathrm{\Box _{\mathrm {P}}}}}p_3$ is set to true. However, we also know that, because  is set to false, the clause will not be satisfied solely by ${{\mathrm{\Box _{\mathrm {P}}}}}p_3$ in all possible worlds of a satisfying model. This indicates that solving the formula using the backdoor will not be as simple as it was for the propositional satisfiability problem, where it was sufficient to enumerate all assignments of the backdoor set and solve the reduced formula. Nevertheless, as we will show in Sect. 5.1 our backdoor sets can still be used for the efficient evaluation of LTL formulas.

To exploit backdoor sets to obtain efficient fpt-algorithms for LTL one needs to accomplish two tasks: first, one needs to find a small backdoor set, and then one needs to show how the backdoor set can be exploited to efficiently evaluate the formula. This leads to the following problem definitions for every class ${\mathcal {C}}$ of formulas and set of operators ${\mathcal {O}}$.

Problem: $\mathrm {Eval}^{{\mathcal {O}}}({\mathcal {C}})$ — Backdoor evaluation to .
Input:
formula $\phi$, strong $({\mathcal {C}}, {\mathcal {O}})$-backdoor *X*.
Parameter: |*X*|.
Question: Is $\phi$ satisfiable?

Problem: $\mathrm {Detect}^{{\mathcal {O}}}({\mathcal {C}})$ — Backdoor detection to .
Input:
formula $$\phi $$, integer $$k\in \mathbb N$$.
Parameter: *k*.
Task: Find a strong $({\mathcal {C}},{\mathcal {O}})$-backdoor of size $\le k$ if one exists.

Of course, this approach is only meaningful if one considers target classes that have polynomial time solvable satisfiability problems. Artale et al. have shown [2] that satisfiability for  and  are solvable in $\mathsf {P}$. Adding ${{\mathrm{\Box _{\mathrm {F}}}}},{{\mathrm{\Box _{\mathrm {P}}}}}$ to the set of allowed operators makes the $\textsc {krom}$ fragment $\mathsf {NP}$-complete whereas for $\textsc {horn}$ formulas the problem stays in $\mathsf {P}$. Accordingly, we will consider in the following only $\textsc {krom}$ and $\textsc {horn}$ formulas. Moreover, note that when considering arbitrary CNF formulas instead of $\textsc {horn}$ or $\textsc {krom}$ formulas, then  is known to be NP-complete for any (even empty) subset  [2].

## Backdoor Set Detection

In this section, we show that finding strong $\mathcal {C}$-backdoor sets (under the parameter size of the set) is fixed-parameter tractable if $\mathcal {C}$ is either $\textsc {horn}$ or $\textsc {krom}$. The algorithms that we will present are very similar to the algorithms that are known for the detection of strong backdoors for propositional CNF formulas [18].

We first show how to deal with the fact that we only need to consider consistent assignments. The following observation is easily witnessed by the fact that if one of ${{\mathrm{\Box _{\mathrm {P}}}}}x,{{\mathrm{\Box _{\mathrm {F}}}}}x, x$ does not hold then  is true.

### Observation 1

Let  be an  formula. Then any clause *C* of $\varPhi$ containing  and (at least) one of $\Box _P x$, $\Box _F x$ or *x* for some variable $x\in \mathrm {Vars}(\phi )$ is tautological and can be removed from $\varPhi$ (without changing the satisfiability of $\phi$).

Observe that the tautological clauses above are exactly the clauses that are satisfied by every consistent assignment. It follows that once these clauses are removed from the formula, it holds that for every clause *C* of $\phi$ there is a consistent assignment $\theta$ such that *C* is not satisfied by $\theta$.

### Theorem 5

For every  and ${\mathcal {C}}\in \{\textsc {horn},\textsc {krom}\}$ the problem $\mathrm {Detect}^{\mathcal {O}}({\mathcal {C}})$ is in $\mathsf {FPT}$.

### Proof

Let . We will reduce $\mathrm {Detect}^{\mathcal {O}}(\textsc {horn})$ to the problem $\mathrm {VertexCover}$ which is well-known to be fixed-parameter tractable (parameterized by the solution size) and which can actually be solved very efficiently in time $O(1.2738^k+kn)$ [6], where *k* is the size of the vertex cover and *n* the number of vertices in the input graph. Recall that given an undirected graph *G* and an integer *k*, $\mathrm {VertexCover}$ asks whether there is a subset $C \subseteq V(G)$ of size at most *k* (which is called a vertex cover of *G*) such that $C \cap e \ne \emptyset$ for every $e \in E(G)$. Given an  formula , we will construct an undirected graph *G* such that $\phi$ has a strong $\textsc {horn}$-backdoor of size at most *k* if and only if *G* has a vertex cover of size at most *k*. The graph *G* has vertex set $\mathrm {Vars}(\phi )$ and there is an edge between two vertices *x* and *y* in *G* if and only if there is a clause that contains at least two literals from $\{x,y\} \cup \{\,Ox, Oy \mid O \in \mathcal {O}\,\}$. Note that if $x=y$, the graph *G* contains a self-loop. We claim that a set $X \subseteq \mathrm {Vars}(\phi )$ is a strong $\textsc {horn}$-backdoor if and only if *X* is a vertex cover of *G*.

Towards showing the forward direction, let $X \subseteq \mathrm {Vars}(\phi )$ be a strong $\textsc {horn}$-backdoor set of $\phi$. We claim that *X* is also a vertex cover of *G*. Suppose for a contradiction that *X* is not a vertex cover of *G*, i.e., there is an edge $\{x,y\} \in E(G)$ such that $X \cap \{x,y\}=\emptyset$. Because $\{x,y\} \in E(G)$, we obtain that there is a clause *C* in $\varPhi$ that contains at least two literals from $\{x,y\} \cup \{\,Ox,Oy \mid O \in \mathcal {O}\,\}$. Moreover, because of Observation 1 there is a consistent assignment $\theta :X\cup \{\,O x\mid x\in X \wedge O \in \mathcal {O}\}\rightarrow \{0,1\}$ that falsifies all literals of *C* over variables in *X*. Consequently, $\phi [\theta ]$ contains a sub-clause of *C* that still contains at least two literals from $\{x,y\} \cup \{\,Ox,Oy \mid O \in \mathcal {O}\,\}$. As a reason for this, $\phi [\theta ] \notin \textsc {horn}$, contradicting our assumption that *X* is a strong $\textsc {horn}$-backdoor set of $\phi$.

Towards showing the reverse direction, let $X \subseteq V(G)$ be a vertex cover of *G*. We claim that *X* is also a strong $\textsc {horn}$-backdoor of $\phi$. Suppose for a contradiction that this is not the case, then there is an (consistent) assignment $\theta :X\cup \{O x\mid x\in X \wedge O \in \mathcal {O}\}\rightarrow \{0,1\}$ and a clause *C* in $\phi [\theta ]$ containing two positive literals say over variables *x* and *y*. We obtain that *C* contains at least two positive literals from $\{x,y\} \cup \{\,Ox,Oy \mid O \in \mathcal {O}\,\}$ and consequently *G* contains the edge $\{x,y\}$, contradicting our assumption that *X* is a vertex cover of *G*.

Now we will reduce $\mathrm {Detect}^{\mathcal {O}}(\textsc {krom})$ to the 3-HittingSet problem, which is well-known to be fixed-parameter tractable (parameterized by the solution size) [1]. Recall that given a universe *U*, a family $\mathcal {F}$ of subsets of *U* of size at most three, and an integer *k*, 3-HittingSet asks whether there is a subset $S \subseteq U$ of size at most *k* (which is called a hitting set of $\mathcal {F}$) such that $S \cap F \ne \emptyset$ for every $F \in \mathcal {F}$. Given an  formula , we will construct a family $\mathcal {F}$ of subsets (of size at most three) of a universe *U* such that $\phi$ has a strong $\textsc {krom}$-backdoor of size at most *k* if and only if $\mathcal {F}$ has a hitting set of size at most *k*. The universe *U* is equal to $\mathrm {Vars}(\phi )$ and $\mathcal {F}$ contains the set $\mathrm {Vars}(C)$ for every set *C* of exactly three literals contained in some clause of $\varPhi$. We claim that a set $X \subseteq \mathrm {Vars}(\phi )$ is a strong $\textsc {krom}$-backdoor if and only if *X* is a hitting set of $\mathcal {F}$.

Towards showing the forward direction, let $X \subseteq \mathrm {Vars}(\phi )$ be a strong $\textsc {krom}$-backdoor set of $\phi$ and suppose for a contradiction that there is a set $F \in \mathcal {F}$ such that $X \cap F=\emptyset$. It follows from the construction of $\mathcal {F}$ that $\varPhi$ contains a clause *C* containing at least three literals over the variables in *F*. Moreover, because of Observation 1 there is a consistent assignment $\theta :X\cup \{\,O x\mid x\in X \wedge O \in \mathcal {O}\}\rightarrow \{0,1\}$ that falsifies all literals of *C* over variables in *X*. Consequently, $\phi [\theta ]$ contains a sub-clause of *C* that still contains at least three literals over the variables in *F*. As a result, $\phi [\theta ]\notin \textsc {krom}$, contradicting our assumption that *X* is a strong $\textsc {krom}$-backdoor set of $\phi$.

Towards showing the reverse direction, let $X \subseteq U$ be a hitting set of $\mathcal {F}$ and suppose for contradiction that there is an (consistent) assignment $\theta :X\cup \{O x\mid x\in X\wedge O \in \mathcal {O}\}\rightarrow \{0,1\}$ and a clause *C* in $\phi [\theta ]$ containing at least three literals. Let $C'$ be a set of at exactly three literals from *C*. It follows from the construction of $\mathcal {F}$, that $\mathcal {F}$ contains the set $\mathrm {Vars}(C')$, however, $\mathrm {Vars}(C') \cap X=\emptyset$ contradicting our assumption that *X* is a hitting set of *G*.$\square$

Having shown that the detection problem is fixed-parameter tractable, we now proceed to the backdoor set evaluation problem. We begin by investigating this problem for the class $\textsc {horn}$ and show that the problem lies in $\mathsf {FPT}$.

## Backdoor Set Evaluation

### Formulas Using only the Always Operator

We showed in the previous section that strong backdoors can be found to the classes $\textsc {horn}$ and $\textsc {krom}$ in $\mathsf {FPT}$ time. In fact, this result holds independently of the considered temporal operators. In this section, we will consider the question of efficiently *using* a backdoor set to decide the satisfiability of a formula in the case of formulas restricted to the  operator. We will show that this problem is in $\mathsf {FPT}$ for the class of $\textsc {horn}$ formulas but not for $\textsc {krom}$ formulas. Our fixed-parameter tractability result for $\textsc {horn}$ formulas largely depends on the special semantics of formulas restricted to the  operators. Consequently, we will start by stating some properties of these formulas necessary to obtain our tractability result.

Let $\mathfrak {M}=(\mathbb Z,<,V)$ be a temporal interpretation. We denote by $\mathrm {Vars}(\mathfrak {M})$ the set of propositions (in the following referred to as variables) for which *V* is defined. For a set of variables $X \subseteq \mathrm {Vars}(\mathfrak {M})$, we denote by $\mathfrak {M}_{|X}$ the *projection* of $\mathfrak {M}$ onto *X*, i.e., the temporal interpretation $\mathfrak {M}_{|X}=(\mathbb Z,<,V_{|X})$, where $V_{|X}$ is only defined for the variables in *X* and $V_{|X}(x)=V(x)$ for every $x \in X$. For an integer *z*, we denote by $\mathbf{A}(\mathfrak {M},z)$ the assignment $\theta :\mathrm {Vars}(\mathfrak {M})\rightarrow \{0,1\}$ holding at world *z* in $\mathfrak {M}$, i.e., $\theta (v)=1$ if and only if $z \in \mathfrak {M}(v)$ for every $v \in \mathrm {Vars}(\mathfrak {M})$. Moreover, for a set of worlds $Z \subseteq \mathbb Z$ we denote by $\mathbf{A}(\mathfrak {M},Z)$ the set of all assignments occurring in some world in *Z* of $\mathfrak {M}$, i.e., $\mathbf{A}(\mathfrak {M},Z)\mathrel {\mathop :}=\{\,\mathbf{A}(\mathfrak {M},z) \mid z \in Z \,\}$. We also set $\mathbf{A}(\mathfrak {M})$ to be $\mathbf{A}(\mathfrak {M},\mathbb Z)$. For an assignment $\theta :X \rightarrow \{0,1\}$, we denote by $\mathbf{W}(\mathfrak {M},\theta )$ the set of all worlds $z \in \mathbb Z$ of $\mathfrak {M}$ such that $\mathbf{A}(\mathfrak {M},z)$ is equal to $\theta$ on all variables in *X*.

**Table 4:** An example for the notions $\mathbf{G}(\mathbb {A},V)$ and $\mathbf{G}(\mathbb {A},V,\theta )$

	$\mathbb {A}$				$\theta$		$\mathbf{G}(\mathbb {A},V,\theta )(v_i)$
	$\alpha _1$	$\alpha _2$	$\alpha _3$	$\alpha _4$			
$v_1$	0	1	0	1	1	0	1
$v_2$	1	1	1	1	0	1	0
$v_3$	1	1	0	0	1	0	1
$v_4$	1	1	1	1	0	1	0

Let . We denote by $\mathbf{CNF}(\varPhi )$ the propositional CNF formula obtained from $\varPhi$ after replacing each occurrence of  in $\varPhi$ with the same fresh propositional variable (with the same name). For instance,  is replaced by , where  is a fresh propositional variable. For a set of variables *V* and a set of assignments $\mathbb {A}$ of the variables in *V*, we denote by  the assignment defined by setting  if and only if $\alpha (v)=1$ for every $\alpha \in \mathbb {A}$. Moreover, if $\theta :V \rightarrow \{0,1\}$ is an assignment of the variables in *V*, we denote by $\mathbf{G}(\mathbb {A},V,\theta )$ the assignment defined by setting $\mathbf{G}(\mathbb {A},V,\theta )(v)=\theta (v)$ and  for every $v \in V$. An example for these notions is given in Table 4. For a set $\mathbb {A}$ of assignments over *V* and an assignment $\theta :V' \rightarrow \{0,1\}$ with $V' \subseteq V$, we denote by $\mathbb {A}(\theta )$ the set of all assignments $\alpha \in \mathbb {A}$ such that $\alpha (v)=\theta (v)$ for every $v \in V'$.

For a set $\mathbb {A}$ of assignments over some variables *V* and a subset $V' \subseteq V$, we denote by $\mathbb {A}|_{V'}$ the *projection* of $\mathbb {A}$ onto $V'$, i.e., the set of assignments $\alpha \in \mathbb {A}$ restricted to the variables in $V'$.

Intuitively the next lemma describes the translation of a temporal model into separate satisfiability checks for propositional formulas.

#### Lemma 6

Let . Then, $\varphi$ is satisfiable if and only if there is a set $\mathbb {A}$ of assignments of the variables in $\varphi$ and an assignment $\alpha _0 \in \mathbb {A}$ such that: $\alpha _0$ satisfies $\varPsi$ and for every assignment $\alpha \in \mathbb {A}$ it holds that $\mathbf{G}(\mathbb {A},\mathrm {Vars}(\varphi ),\alpha )$ satisfies the propositional formula $\mathbf{CNF}(\varPhi )$.

#### Proof

Towards showing the forward direction assume that  is satisfiable and let $\mathfrak {M}$ be a temporal interpretation witnessing this. It is easy to check from the definition that the set of assignments $\mathbb {A}\mathrel {\mathop :}=\mathbf{A}(\mathfrak {M})$ together with the assignment $\alpha _0\mathrel {\mathop :}=\mathbf{A}(\mathfrak {M},0)$ satisfy the conditions of the lemma.

Towards showing the reverse direction assume that $\mathbb {A}\mathrel {\mathop :}=\{\alpha _0,\cdots ,\alpha _{|\mathbb {A}|-1}\}$ is as given in the statement of the lemma. We claim that the temporal interpretation $\mathfrak {M}$ defined below satisfies the formula $\varphi$. Let $\mathbb Z_{<0}$ be the set of all integers smaller than 0 and let $\mathbb Z_{>|\mathbb {A}|}$ be the set of all integers greater than $|\mathbb {A}|$. Then for every variable $v \in \mathrm {Vars}(\varphi )$, the set $\mathfrak {M}(v)$ contains the set $\{\,z \mid \alpha _z(v)=1 \wedge 0 \le z \le |\mathbb {A}|\,\}$. Moreover, if $\alpha _0(v)=1$, $\mathfrak {M}(v)$ also contains the set $\mathbb Z_{<0}$ and if $\alpha _{|\mathbb {A}|}(v)=1$, $\mathfrak {M}(v)$ additionally contains the set $\mathbb Z_{>|\mathbb {A}|}$. It is easy to verify that $\mathfrak {M},0 \models \varphi$. $\square$

Informally, the following lemma shows that for deciding the satisfiability of an  formula, we only need to consider sets of assignments $\mathbb {A}$, whose size is linear (instead of exponential) in the number of variables.

#### Lemma 7

Let  and $X \subseteq \mathrm {Vars}(\varphi )$. Then $\varphi$ is satisfiable if and only if there is a set $\varTheta$ of assignments of the variables in *X*, an assignment $\theta _0 \in \varTheta$, a set $\mathbb {A}$ of assignments of the variables in $\mathrm {Vars}(\varphi )$, and an assignment $\alpha _0 \in \mathbb {A}$ such that:

1. the set $\varTheta$ is equal to $\mathbb {A}_{|X}$,
2. the assignment $\theta _0$ is equal to $\alpha _0|_X$,
3. $\mathbb {A}$ and $\alpha _0$ satisfy the conditions stated in Lemma 6, and
4. $$|\mathbb {A}(\theta )| \le |\mathrm {Vars}(\varphi )\setminus X|+1$$ for every $$\theta \in \varTheta $$.

#### Proof

Note that the reverse direction follows immediately from Lemma 6, because the existence of the set of assignments $\mathbb {A}$ and the assignment $\alpha _0$ satisfying condition (C3) imply the satisfiability of $\varphi$.

Towards showing the forward direction assume that $\varphi$ is satisfiable. Because of Lemma 6 there is a set $\mathbb {A}$ of assignments of the variables in $\varphi$ and an assignment $\alpha _0 \in \mathbb {A}$ that satisfy the conditions of Lemma 6. Let $\varTheta$ be equal to $\mathbb {A}_{|X}$ and $\theta _0$ be equal to $\alpha _0|_X$. Observe that setting $\varTheta$ and $\theta _0$ in this way already satisfies (C1) to (C3). We will show that there is a subset of $\mathbb {A}$ that still satisfies (C1)–(C3) and additionally (C4). Towards showing this consider any subset $\mathbb {A}'$ of $\mathbb {A}$ that satisfies the following three conditions: (1) $\alpha _0 \in \mathbb {A}'$, (2) for every $\theta \in \varTheta$ it holds that $\mathbb {A}'(\theta )\ne \emptyset$, and (3) for every variable *v* of $\varphi$ and every $b \in \{0,1\}$ it holds that there is an assignment $\alpha \in \mathbb {A}$ with $\alpha (v)=i$ if and only if there is an assignment $\alpha ' \in \mathbb {A}'$ with $\alpha '(v)=i$. Note that conditions (1) and (2) ensure that $\mathbb {A}'$ satisfies (C1) and (C2) and condition (3) ensures (C3). Accordingly, any subset $\mathbb {A}'$ satisfying conditions (1)–(3) still satisfies (C1)–(C3). It remains to show how to obtain such a subset $\mathbb {A}'$ that additionally satisfies (C4). We define $\mathbb {A}'$ as follows. Let $\mathbb {A}_0'$ be a subset of $\mathbb {A}$ containing $\alpha _0$ as well as one arbitrary assignment $\alpha \in \mathbb {A}(\theta )$ for every $\theta \in \varTheta$. Note that $\mathbb {A}_0'$ already satisfies conditions (1) and (2) as well as condition (3) for every variable $v \in X$. Observe furthermore that if there is a variable *v* of $\varphi$ such that condition (3) is violated by $\mathbb {A}_0'$ then it is sufficient to add at most one additional assignment to $\mathbb {A}_0'$ in order to satisfy condition (3) for *v*. Let $\mathbb {A}'$ be obtained from $\mathbb {A}_0'$ by adding (at most $|\mathrm {Vars}(\varphi )\setminus X|$) assignments in order to ensure condition (3) for every variable $v \in \mathrm {Vars}(\varphi )\setminus X$. Then $\mathbb {A}'$ satisfies the conditions of the lemma. $\square$

We are now ready to show the tractability of the evaluation of strong $\textsc {horn}$-backdoor sets.

#### Theorem 8

 is in $$\mathsf {FPT}$$.

#### Proof

Let  and let $X \subseteq \mathrm {Vars}(\varphi )$ be a strong $\textsc {horn}$-backdoor of $\varphi$. The main idea of the algorithm is as follows: For every set $\varTheta$ of assignments of the variables in *X* and every $\theta _0 \in \varTheta$, we will construct a propositional $\textsc {horn}$-formula $F_{\varTheta ,\theta _0}$, which is satisfiable if and only if there is a set $\mathbb {A}$ of assignments of the variables in $\mathrm {Vars}(\varphi )$ and an assignment $\alpha _0 \in \mathbb {A}$ satisfying the conditions of Lemma 7. It then follows from Lemma 7 that $\varphi$ is satisfiable if and only if there is such a set $\varTheta$ of assignments and an assignment $\theta _0 \in \varTheta$ for which $F_{\varTheta ,\theta _0}$ is satisfiable. Because there are at most $2^{2^{|X|}}$ such sets $\varTheta$ and at most $2^{|X|}$ such assignments $\theta _0$ and for each of these sets the formula $F_{\varTheta ,\theta _0}$ is a $\textsc {horn}$-formula, it follows that checking whether there are $\varTheta$ and $\theta _0$ such that the formula $F_{\varTheta ,\theta _0}$ is satisfied (and as a result decide the satisfiability of $\varphi$) can be done in time $O(2^{2^{|X|}}\cdot 2^{|X|}\cdot |F_{\varTheta ,\theta _0}|)$. Since we will show below that the length of the formula $F_{\varTheta ,\theta _0}$ can be bounded by an (exponential) function of |*X*| times a polynomial in the input size, i.e., the length of the formula $\varphi$, this implies that  is in $\mathsf {FPT}$.

The remainder of the proof is devoted to the construction of the formula $F_{\varTheta ,\theta _0}$ for a fixed set of assignments $\varTheta$ and a fixed assignment $\theta _0 \in \varTheta$ (and to show that it enforces the conditions of Lemma 7).

Let $R\mathrel {\mathop :}=\mathrm {Vars}(\varphi )\setminus X$ and $r\mathrel {\mathop :}=|R|+1$. For a propositional formula *F*, a subset $V \subseteq \mathrm {Vars}(F)$, an integer *i* and a label *s*, we denote by $\mathbf{copy}(F,V,i,s)$ the propositional formula obtained from *F* after replacing each occurrence of a variable $v \in V$ with a novel variable $v^i_s$. We need the following auxiliary formulas. For every $\theta \in \varTheta \setminus \{\theta _0\}$, let $F_{\varTheta ,\theta _0}^\theta$ be the formula (where the notation $\varPhi [\mathbf{G}(\varTheta ,X,\theta )]$ refers to the formula that is obtained after applying the assignment $\mathbf{G}(\varTheta ,X,\theta )$ in the usual sense, that is, removing satisfied clauses and deleting falsified literals):

$$\begin{aligned} \bigwedge _{1\le i \le r}\mathbf{copy}(\mathbf{CNF}(\varPhi [\mathbf{G}(\varTheta ,X,\theta )]),R,i,\theta ). \end{aligned}$$

Moreover, let $F_{\varTheta ,\theta _0}^{\theta _0}$ be the formula:

$$\begin{aligned} \mathbf{copy}(\varPsi [\theta _0] \wedge \mathbf{CNF}(\varPhi [\mathbf{G}(\varTheta ,X,\theta _0)]),R,1,\theta _0) \,\wedge \\ \bigwedge _{2\le i \le r}\mathbf{copy}(\mathbf{CNF}(\varPhi [\mathbf{G}(\varTheta ,X,\theta _0)]),R,i,\theta _0). \end{aligned}$$

Observe that because *X* is a strong $\textsc {horn}$-backdoor set (and the formula $\varPsi$ only consists of unit clauses), it holds that the formula $F_{\varTheta ,\theta _0}^\theta$ is $\textsc {horn}$ for every $\theta \in \varTheta$. We also need the propositional formula $F_{cons }$ that enforces the consistency between the propositional variables  and the variables in $\{\,x_\theta ^i \mid \theta \in \varTheta \wedge 1\le i \le r \,\}$ for every $x \in \mathrm {Vars}(\varphi )\setminus X$. The formula $F_{cons }$ consists of the following clauses: for every $\theta \in \varTheta$, *i* with $1 \le i \le r$, and $v \in R$, the clause  and for every $v \in R$ the clauseObserve that $F_{cons }$ is a $\textsc {horn}$ formula.

Finally the formula $F_{\varTheta ,\theta _0}$ is defined as: $\bigwedge _{\theta \in \varTheta }F_{\varTheta ,\theta _0}^\theta \wedge F_{cons }.$

Note that $F_{\varTheta ,\theta _0}$ is $\textsc {horn}$ and the length of $F_{\varTheta ,\theta _0}$ is at most

$$\begin{aligned} |F_{\varTheta ,\theta _0}|&\le \sum _{\theta \in \varTheta }|F_{\varTheta ,\theta _0}^{\theta }|+|F_{cons }|\\&\le 2^{|X|}(|\mathrm {Vars}(\varphi )\setminus X|+1)(|\varPhi |+|\varPsi |)+2\cdot 2^{|X|}\cdot (|\mathrm {Vars}(\varphi )\setminus X|+1)^2 \end{aligned}$$

and consequently bounded by a function of |*X*| times a polynomial in the input size. It is now relatively straightforward to verify that $F_{\varTheta ,\theta }$ is satisfiable if and only if there is a set $\mathbb {A}$ of assignments of the variables in $\mathrm {Vars}(\varphi )$ and an assignment $\alpha _0 \in \mathbb {A}$ satisfying the conditions of Lemma 7. Informally, for every $\theta \in \varTheta$, each of the *r* copies of the formula $\mathbf{CNF}(\varPhi [\mathbf{G}(\varTheta ,X,\theta )])$ represent one of the at most *r* assignments in $\mathbb {A}(\theta )$, the formula $F_{\varTheta ,\theta _0}^{\theta _0}$ ensures (among other things) that the assignment chosen for $\alpha _0$ satisfies $\varPsi$ and the formula $F_{cons }$ ensures that the “global assignments” represented by the propositional variables  are consistent with the set of local assignments in $\mathbb {A}$ represented by the variables in $\{\,x_\theta ^i \mid \theta \in \varTheta \wedge 1\le i \le r \,\}$ for every $x \in \mathrm {Vars}(\varphi )\setminus X$.

$$\square $$

Surprisingly, the next result will show that $\textsc {krom}$ formulas turn out to be quite challenging. Backdoor set evaluation of this class of formulas is proved to be $\mathsf {paraNP}$-complete which witnesses an intractability degree in the parameterized sense.

#### Theorem 9

 is $\mathsf {paraNP}$-complete (the $\mathsf {NP}$-completeness already holds for backdoor sets of size two).

#### Proof

The membership in $\mathsf {paraNP}$ follows because the satisfiability of  can be decided in $\mathsf {NP}$ [2, Table 1].

We show $\mathsf {paraNP}$-hardness of  by giving a polynomial time reduction from the $\mathsf {NP}$-hard problem $\mathrm {3COL}$ to  for backdoors of size two. In $\mathrm {3COL}$ one asks whether a given input graph $G=(V,E)$ has a coloring $f :V(G)\rightarrow \{1,2,3\}$ of its vertices with at most three colors such that $f(v)\ne f(u)$ for every edge $\{u,v\}$ of *G*. Given such a graph $G=(V,E)$, we will construct an  formula , which has a strong $\textsc {krom}$-backdoor *B* of size two, such that the graph *G* has a 3-coloring if and only if $\phi$ is satisfiable.

For the remainder we will assume that there exists an arbitrary but fixed ordering of the vertices $V(G)=\{v_1,\cdots ,v_n\}$. Further for the construction we assume w.l.o.g. that any undirected edge $e=\{v_i,v_j\}\in E$ follows this ordering, i.e., $i<j$. The formula $\phi$ contains the following variables:

1. The variables $b_1$ and $b_2$. These variables make up the backdoor set *B*, i.e., $B\mathrel {\mathop :}=\{b_1,b_2\}$.
2. For every *i* with $1 \le i \le n$, the variable $v_i$.
3. For every $e=\{v_i,v_j\} \in E(G)$ with $1 \le i,j \le n$ the variables $e_{i,j}^{b_1b_2}$, $e_{i,j}^{{\bar{b}}_1b_2}$, and $e_{i,j}^{b_1{\bar{b}}_2}$.

We set $\varPsi$ to be the empty formula and the formula $\varPhi$ contains the following clauses:

1. For every *i* with $1 \le i \le n$, the clause . Informally, this clause ensures that $v_i$ has to be false at least at one world, which will later be used to assign a color to the vertex $v_i$ of *G*. Observe that the clause is $\textsc {krom}$.
2. For every $e=\{v_i,v_j\} \in E(G)$ with $1 \le i,j \le n$, the clauses , , and  as well as the clauses , , and . Observe that all of these clauses are $\textsc {krom}$ after deleting the variables in *B*.
3. The clause $\lnot b_1 \vee \lnot b_2$. Informally, this clause excludes the color represented by setting $b_1$ and $b_2$ to true. Observe that the clause is $\textsc {krom}$.

It follows from the definition of $\phi$ that  for every assignment $\theta$ of the variables in *B*. As a consequence, *B* is a strong $\textsc {krom}$-backdoor of size two of $\phi$ as required. Moreover, since $\phi$ can be constructed in polynomial time, it only remains to show that *G* has a 3-coloring if and only if $\phi$ is satisfiable.

**Table 5:** Given a graph $G=(\{v_1,v_2,v_3\},\{\{v_1,v_2\},\{v_1,v_3\},\{v_2,v_3\}\})$ together with a 3-coloring $f(v_i)=i$ for $1\le i\le 3,$ leads to the depicted temporal interpretation $\mathfrak {M}$ satisfying $\mathfrak {M}\models \phi$ given as a table

	$b_1$	$b_2$	$v_1$	$v_2$	$v_3$	$e_{1,2}^{b_1b_2}$	$e_{1,2}^{\bar{b}_1b_2}$	$e_{1,2}^{b_1\bar{b}_2}$	$e_{1,3}^{b_1b_2}$	$e_{1,3}^{\bar{b}_1b_2}$	$e_{1,3}^{b_1\bar{b}_2}$	$e_{2,3}^{b_1b_2}$	$e_{2,3}^{\bar{b}_1b_2}$	$e_{2,3}^{b_1\bar{b}_2}$
1	0	0	0	1	1	1	0	$*$	1	$*$	0	$*$	1	0
2	1	0	1	0	1	1	0	$*$	1	$*$	0	$*$	1	0
3	0	1	1	1	0	1	0	$*$	1	$*$	0	$*$	1	0

Each row of the table corresponds to a world as indicated by the first column of the table. Each column represents the assignments of a variable as indicated in the first row. A “$*$” indicates that the assignment is not fixed, i.e., the assignment does not influence whether $\mathfrak {M}\models \phi$

Towards showing the forward direction assume that *G* has a 3-coloring and let $f :V(G) \rightarrow \{1,2,3\}$ be such a 3-coloring for *G*. We will show that $\phi$ is satisfiable by constructing a temporal interpretation $\mathfrak {M}$ such that $\mathfrak {M}\models \phi$. The interpretation $\mathfrak {M}$ is defined as follows:

- For every *i* with $1 \le i \le n$, we set $\mathfrak {M}(v_i)=\mathbb Z\setminus \{f(v_i)\}$.
- We set $\mathfrak {M}(b_1)=\{2\}$ and $\mathfrak {M}(b_2)=\{3\}$.
- For every $$e=\{v_i,v_j\} \in E(G)$$:
  - if $f(v_i)=1$ set $\mathfrak {M}(e_{i,j}^{b_1b_2})=\mathbb Z$, else set $\mathfrak {M}(e_{i,j}^{b_1b_2})=\emptyset$.
  - if $f(v_i)=2$ set $\mathfrak {M}(e_{i,j}^{\bar{b}_1b_2})=\mathbb Z$, else set $\mathfrak {M}(e_{i,j}^{\bar{b}_1b_2})=\emptyset$.
  - if $f(v_i)=3$ set $\mathfrak {M}(e_{i,j}^{b_1\bar{b}_2})=\mathbb Z$, else set $\mathfrak {M}(e_{i,j}^{b_1\bar{b}_2})=\emptyset$.

An example for such a temporal interpretation resulting for a simple graph is illustrated in Table 5. Towards showing that $\mathfrak {M}\models \phi$, we consider the different types of clauses given in (C1)–(C3).

- The clauses in (C1) hold because $\mathfrak {M}, f(v_i) \not \models v_i$ for every *i* with $1 \le i \le n$.
- For every $e=\{v_i,v_j\} \in E(G)$, we have to show that the clauses given in (C2) are satisfied for every world. Because *f* is a 3-coloring of *G*, we obtain that $f(v_i)\ne f(v_j)$. W.l.o.g. we assume in the following that $f(v_i)=1$ and $f(v_j)=2$. We first consider the clauses given in (C2) containing $v_i$. Because $\mathfrak {M}(v_i)=\mathbb Z\setminus \{1\}$, it only remains to consider the world 1. In this world $b_1$ and $b_2$ are false. It follows that all clauses containing either $\lnot b_1$ or $\lnot b_2$ are satisfied in this world. As a reason for this, it only remains to consider clauses of the form . But these are satisfied because $f(v_i)=1$ implies that $\mathfrak {M}(e_{i,j}^{b_1b_2})=\mathbb Z$.
  Consider now the clauses given in (C2) that contain $v_j$. Using the same argumentation as used above for $v_i$, we obtain that we only need to consider world 2 and moreover we only need to consider clauses of the form . Because $f(v_i)=1$, we obtain that $\mathfrak {M}(e_{i,j}^{\bar{b}_1b_2})=\emptyset$, which implies that these clauses are also satisfied.
- The clause $\lnot b_1 \vee \lnot b_2$ is trivially satisfied, because there is no world in which $b_1$ and $b_2$ hold simultaneously.

Towards showing the reverse direction assume that $\phi$ is satisfiable and let $\mathfrak {M}$ be a temporal interpretation witnessing this. First note that because of the clauses added by C1, it holds that $\mathfrak {M}(v_i)\ne \mathbb Z$ for every *i* with $1\le i \le n$. Let $w :V(G) \rightarrow \mathbb Z$ be defined such that for every *i* with $1 \le i \le n$, $w(v_i)$ is an arbitrary world in $\mathbb Z\setminus \mathfrak {M}(v_i)$. We define $f :V(G) \rightarrow \{1,2,3\}$ by setting:

- $$f(v_i)=1$$ if $$\mathfrak {M},w(v_i) \not \models b_1 \vee b_2$$,
- $$f(v_i)=2$$ if $$\mathfrak {M},w(v_i) \not \models \lnot b_1 \vee b_2$$, and
- $$f(v_i)=3$$ if $$\mathfrak {M},w(v_i) \not \models b_1 \vee \lnot b_2$$.

Note that because of the clause added by (C3), *f* assigns exactly one color to every vertex $v_i$ of *G*. We claim that *f* is a 3-coloring of *G*. To show this it suffices to show that for every $e=\{v_i,v_j\}\in E(G)$, it holds that $f(v_i)\ne f(v_j)$. Assume for a contradiction that this is not the case, i.e., there is an edge $e=\{v_i,v_j\}\in E(G)$ such that $f(v_i)=f(v_j)$. W.l.o.g. assume furthermore that $f(v_i)=f(v_j)=1$. Consider the clause  (which was added by C2). Then, because of the definition of *w* and *f*, we obtain that $\mathfrak {M}, w(v_i) \not \models v_i \vee b_1 \vee b_2$. It follows that . Consider now the clause  (which was added by C2). Then, again because of the choice of *w* and *f*, we obtain that $\mathfrak {M},w(v_j)\not \models v_j \vee b_1 \vee b_2$. As a consequence,  contradicting . This completes the proof of the theorem. $\square$

### Globally in the Past and Globally in the Future

Now we turn to a more flexible fragment where we can talk about the past as well as about the future and show it is possible to encode $\mathsf {NP}$-complete problems into the $\textsc {horn}$-fragment yielding a $\mathsf {paraNP}$ lower bound.

#### Theorem 10

$\mathrm {Eval}^{\Box _F,\Box _P}(\textsc {horn})$ is $\mathsf {paraNP}$-complete (the $\mathsf {NP}$-completeness already holds for backdoor sets of size four).

#### Proof

The membership in $\mathsf {paraNP}$ follows as the satisfiability of  can be decided in $\mathsf {NP}$ [2, Table 1].

We show $\mathsf {paraNP}$-hardness of $\mathrm {Eval}^{\Box _F,\Box _P}(\textsc {horn})$ by describing a polynomial time reduction again from $\mathrm {3COL}$ to $\mathrm {Eval}^{\Box _F,\Box _P}(\textsc {horn})$ for backdoors of size four. Recall that in $\mathrm {3COL}$ one asks whether a given input graph $G=(V,E)$ has a coloring $f:V(G) \rightarrow \{1,2,3\}$ of its vertices with at most three colors such that $f(v)\ne f(u)$ for every edge $\{u,v\}$ of *G*. Given such a graph $G=(V,E)$, we will construct an  formula , which has a strong $\textsc {horn}$-backdoor *B* of size four, such that the graph *G* has a 3-coloring if and only if $\phi$ is satisfiable.

For the remainder we will assume that $V(G)=\{v_1,\cdots ,v_n\}$ and $E(G)=\{e_1,\cdots ,e_m\}$. The formula $\phi$ contains the following variables:

1. The variables $c_1,c_2,c_3,p_n'$ . These variables make up the backdoor set *B*, i.e., $B\mathrel {\mathop :}=\{c_1,c_2,c_3,p_n'\}$.
2. The variable *s*, which indicates the starting world.
3. For every *i* with $1 \le i \le n$, three variables $v_i^1,v_i^2,v_i^3$.
4. For every *i* with $1 \le i \le n$ the variable $p_i$.

We set $\varPsi$ to be the formula *s* and the formula $\varPhi$ contains the following clauses:

1. The clauses $c_1 \vee c_2 \vee c_3$, $\lnot c_1 \vee \lnot c_2 \vee \lnot c_3$, $c_1 \vee \lnot c_2 \vee \lnot c_3$, $\lnot c_1 \vee \lnot c_2 \vee c_3$, and $\lnot c_1 \vee c_2 \vee \lnot c_3$. Informally, these clauses ensure that in every world it holds that exactly one of the variables $c_1,c_2,c_3$ is true. Note that $c_1 \vee c_2 \vee c_3$ is not $\textsc {horn}$, however, all of its variables are contained in the backdoor set *B*.
2. For every *i* and *c* with $1 \le i \le n$ and $1 \le c \le 3$, the clauses $v_i^c \rightarrow \Box _F v_i^c$ and $v_i^c \rightarrow \Box _P v_i^c$; note that $v_i^c \rightarrow \Box _F v_i^c$ corresponds to the clause $\lnot v_i^c \vee \Box _F v_i^c$. Informally, these clauses ensure that the variable $v_i^c$ either holds in every world or in no world for every *i* and *c* as above. Observe that both of these clauses are $\textsc {horn}$.
3. Informally, the following set of clauses ensures together that for every *i* with $1\le i \le n$, it holds that $p_i$ is true in every world apart from the *i*-th world (where $p_i$ is false). Here, the first world is assumed to be the starting world.
  (C3-1) The clauses $s \rightarrow \lnot p_1$, $s \rightarrow \Box _F p_1$, and $s \rightarrow \Box _P p_1$. Informally, these ensure that $p_1$ is only false in the starting world (and otherwise true).
  (C3-2) The clause $p_i \wedge \Box _F p_i\rightarrow \Box _F p_{i+1}$ for every *i* with $1 \le i < n$. Informally, these clauses (together with the clauses from C3-1) ensure that for every *i* with $2 \le i\le n$, it holds that $p_i$ is true in every world after the *i*-th world.
  (C3-3) The clause $\lnot p_i \rightarrow \lnot \Box _F p_{i+1}$ for every *i* with $1 \le i < n$. Informally, these clauses (together with the clauses from C3-1 and C3-2) ensure that for every *i* with $2 \le i\le n$, it holds that $p_i$ is false at the *i*-th world. Observe that the clauses from C3-1 to C3-3 already ensure that $\lnot p_i \wedge \Box _F p_i$ holds if and only if we are at the *i*-th world of the model for every *i* with $1 \le i \le n$.
  (C3-4) The clauses $\lnot p_n\wedge \Box _F p_n\rightarrow p_n'$ and $\lnot p_n\wedge \Box _F p_n \leftarrow p_n'=\lnot p_n\wedge \Box _F p_n \vee \lnot p_n'=(\lnot p_n \vee \lnot p_n') \wedge (\Box _F p_n \vee \lnot p_n')$. Informally, these clauses (together with the clauses from C3-1 to C3-3) ensure that $p_n'$ only holds in the *n*-th world of the model. Observe that all these clauses are $\textsc {horn}$ after removing the backdoor set variable $p_n'$.
  (C3-5) The clause $p_n' \rightarrow \Box _P p_n$. Informally, this clause (together with the clauses from C3-1 to C3-4) ensures that $p_n$ is only false in the *n*-th world of the model.
  (C3-6) The clause $p_i \wedge \Box _P p_i \rightarrow \Box _P p_{i-1}$ for every *i* with $2 \le i \le n$. Informally, these clauses (together with the clauses from C3-1 to C3-5) ensure that $p_i$ is true before the *i*-th world for every *i* with $2 \le i < n$.
  Observe that all of the above clauses are $\textsc {horn}$ or become $\textsc {horn}$ after removing all variables from *B*. Note furthermore that all the above clauses ensure that $\Box _P p_i \wedge \Box _F p_i$ holds if and only if we are at the *i*-th world of the model for every *i* with $1 \le i \le n$.
4. For every *i* and *j* with $1 \le i \le n$ and $1\le j \le 3$ the clauses $\Box _Fp_i \wedge \Box _P p_i \wedge v_i^j \rightarrow c_j$ and $\Box _F p_i \wedge \Box _P p_i \wedge c_j \rightarrow v_i^j$. Informally, these clauses ensure that in the *i*-th world for every $1 \le i\le n$, the variables $c_1$, $c_2$, $c_3$ are a copy of the variables $v_i^1$, $v_i^2$, $v_i^3$. Observe that all of these clauses are $\textsc {horn}$.
5. For every edge $e=\{v_i,v_j\} \in E(G)$ and every *c* with $1 \le c \le 3$, the clause $\lnot v_i^c \vee \lnot v_j^c$. Informally, these clauses ensure that the 3-partition (of the vertices of *G*) given by the (global) values of the variables $v_1^1$, $v_1^2$, $v_1^3$, $\cdots$, $v_n^1$, $v_n^2$, $v_n^3$ is a valid 3-coloring for *G*. Observe that all of these clauses are $\textsc {horn}$.

It follows from the definition of $\phi$ that  for every assignment $\theta$ of the variables in *B*. Consequently, *B* is a strong $\textsc {horn}$-backdoor of size four of $\phi$ as required. Moreover, since $\phi$ can be constructed in polynomial time, it only remains to show that *G* has a 3-coloring if and only if $\phi$ is satisfiable.

**Fig. 1:**
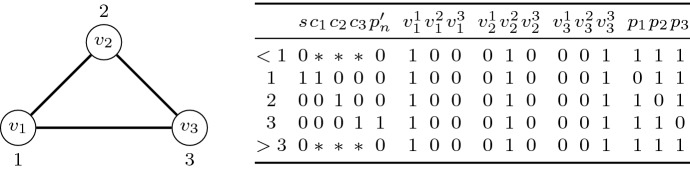
Left: A graph *G* with vertices $v_1$, $v_2$, and $v_3$ together with a 3-coloring given by the numbers above and below respectively of every vertex. Right: A temporal interpretation $\mathfrak {M}$ that corresponds to the given 3-coloring of *G* and satisfies $\mathfrak {M}\models \phi$ given as a table. Each row of the table corresponds to a world (or a set of worlds) as indicated by the first column of the table. Each column represents the assignments of a variable as indicated in the first row. A “$*$” indicates that the assignment is not fixed, i.e., the assignment does not influence whether $\mathfrak {M}\models \phi$

Towards showing the forward direction assume that *G* has a 3-coloring and let $f :V(G) \rightarrow \{1,2,3\}$ be such a 3-coloring for *G*. We will show that $\phi$ is satisfiable by constructing a temporal interpretation $\mathfrak {M}$ such that $\mathfrak {M}\models \phi$. $\mathfrak {M}$ is defined as follows:

- For every *j* with $1\le j \le 3$, we set $\mathfrak {M}(c_j)=\{\,i \mid f(v_i)=j\,\}$.
- We set $$\mathfrak {M}(p_n')=\{n\}$$.
- For every *i* and *c* with $1 \le i \le n$ and $1\le c \le 3$, we set $\mathfrak {M}(v_i^{c})=\mathbb Z$ if $c=f(v_i)$ and otherwise we set $\mathfrak {M}(v_i^c)=\emptyset$.
- For every *i* with $1 \le i \le n$, we set $\mathfrak {M}(p_i)=\mathbb Z\setminus \{i\}$.

An example for such a temporal interpretation resulting for a simple graph is illustrated in Figure 1. It is straightforward (but a little tedious) to verify that $\mathfrak {M}\models \phi$ by considering all the clauses of $\phi$.

Towards showing the reverse direction assume that $\phi$ is satisfiable and let $\mathfrak {M}$ be a temporal interpretation witnessing this. We will start by showing the following series of claims for $\mathfrak {M}$.

1. For every $a \in \mathbb Z$ exactly one of $\mathfrak {M}, a \models c_1$, $\mathfrak {M}, a \models c_2$, and $\mathfrak {M}, a \models c_3$ holds.
2. For every *i*, *c*, *a*, and $a'$ with $1 \le i \le n$, $1 \le c \le 3$, and $a,a' \in \mathbb Z$, it holds that $\mathfrak {M}, a \models v_i^c$ if and only if $\mathfrak {M}, a' \models v_i^c$.
3. For every *i* with $1 \le i \le n$ and every $a \in \mathbb Z$, it holds that $\mathfrak {M}, a \models p_i$ if and only if $a \ne i$.
4. For every *i* and *j* with $1 \le i \le n$ and $1 \le j \le 3$, it holds that $\mathfrak {M}, i \models c_j$ if and only if $\mathfrak {M},i \models v_i^j$.

(M1) holds because of the clauses added by (C1). Towards showing (M2) consider the clauses added by (C2) and assume for a contradiction that there are *i*, *c*, *a*, and $a'$ as in the statement of (M2) such that w.l.o.g. $\mathfrak {M}, a \models v_i^c$ but $\mathfrak {M}, a' \not \models v_i^c$. Then, $a \ne a'$. If $a < a'$, then we obtain a contradiction because of the clause $v_i^c \rightarrow \Box _F v_i^c$ and if on the other hand $a' < a$, we obtain a contradiction to the clause $v_i^c \rightarrow \Box _P v_i^c$. This completes the proof of (M2). Considering the explanations for the clauses the proof of (M3) is now reasonably straightforward, however, for completeness we now provide a detailed proof. We will show (M3) with the help of the following series of claims.

(M3-1) For every $a \in \mathbb Z$ it holds that $\mathfrak {M},a \models p_1$ if and only if $a \ne 1$ (here we assume that 1 is the starting world).
(M3-2) For every *i* and *a* with $1 \le i \le n$, $a \in \mathbb Z$, and $a>i$, it holds that $\mathfrak {M},a \models p_i$.
(M3-3) For every *i* with $1 \le i \le n$, it holds that $\mathfrak {M},i \not \models p_i$.
(M3-4) For every $a \in \mathbb Z$, it holds that $\mathfrak {M},a \models p_n'$ if and only if $a=n$.
(M3-5) For every $a \in \mathbb Z$, it holds that $\mathfrak {M},a \not \models p_n$ if and only if $a=n$.

Because of the clause $s\rightarrow \lnot p_1$ (added by C3-1) and the fact that $s \in \varPsi$, we obtain that $\mathfrak {M},1 \not \models p_1$. Moreover, because of the clauses $s \rightarrow \Box _F p_1$ and $s \rightarrow \Box _P p_1$, we obtain that $\mathfrak {M},a \models p_1$ for every $a \ne 1$. This completes the proof for (M3-1).

We show (M3-2) via induction on *i*. The claim clearly holds for $i=1$ because of (M3-1). Now assume that the claim holds for $p_{i-1}$ and we want to show it for $p_i$. Because of the induction hypothesis, we obtain that $\mathfrak {M},i \models p_{i-1} \wedge \Box _Fp_{i-1}$. Moreover, because $\phi$ contains the clause $p_{i-1} \wedge \Box _F p_{i-1} \rightarrow \Box _F p_i$ (which was added by (C3-2)), we obtain that $\mathfrak {M},i \models \Box _F p_i$. This completes the proof of (M3-2).

We show (M3-3) via induction on *i*. The claim clearly holds for $i=1$ because of (M3-1). Now assume that the claim holds for $p_{i-1}$ and we want to show it for $p_i$. Because of the induction hypothesis, we obtain that $\mathfrak {M},(i-1) \not \models p_{i-1}$. Furthermore, because of (M3-2), we know that $\mathfrak {M},i \models \Box _Fp_{i}$. Since $\phi$ contains the clause $\lnot p_{i-1} \rightarrow \lnot \Box _F p_{i}$ (which was added by (C3-3)), we obtain $\mathfrak {M}, (i-1) \models \lnot \Box _F p_i$, which because $\mathfrak {M},i \models \Box _Fp_{i}$ can only hold if $\mathfrak {M},i \not \models p_i$. This completes the proof of (M3-3).

Towards showing (M3-4), first note that because of (M3-2) and (M3-3), we have that $\mathfrak {M},a \models \lnot p_n \wedge \Box _F p_n$ if and only if $a=n$. Then, because of the clauses (added by C3-4) ensuring that $\lnot p_n \wedge \Box _F p_n \leftrightarrow p_n'$, the same applies to $p_n'$ (instead of $\lnot p_n \wedge \Box _F p_n$). This completes the proof of (M3-4).

It follows from (M3-2) and (M3-3) that (M3-5) holds for every $a \in \mathbb Z$ with $a \ge n$. Moreover, because of (M3-4), we have that $\mathfrak {M}, n \models p_i'$. Because of the clause $p_n' \rightarrow \Box _P p_n$ (which was added by (C3-5)), we obtain $\mathfrak {M},a \models p_n$ for every $a<n$. This completes the proof of (M3-5).

We are now ready to prove (M3). It follows from (M3-2) and (M3-3) that (M3) holds for every *i* and *a* with $a \ge i$. Furthermore, we obtain from (M3-5) that (M3) already holds if $i=n$. We complete the proof of (M3) via an induction on *i* starting from $i=n$. Because of the induction hypothesis, we obtain that $\mathfrak {M},i+1 \models p_{i+1} \wedge \Box _P p_{i+1}$. Accordingly, because of the clause $p_{i+1}\wedge \Box _P p_{i+1}\rightarrow \Box _P p_{i}$ (added by (C3-6)), we obtain that $\mathfrak {M},i+1 \models \Box _P p_i$, which completes the proof of (M3).

Towards showing (M4) first note that it follows from (M3) that $\mathfrak {M}, i \models \Box _F p_i \wedge \Box _P p_i$. Now suppose that there are *i* and *j* such that either $\mathfrak {M}, i \models c_j$ but $\mathfrak {M},i \not \models v_i^j$ or $\mathfrak {M}, i \not \models c_j$ but $\mathfrak {M},i \models v_i^j$. In the former case, consider the clause $\Box _F p_i \wedge \Box _P p_i \wedge c_j \rightarrow v_i^j$ (which was added by (C4)). Since $\mathfrak {M}, i \models \Box _F p_i \wedge \Box _P p_i$, we obtain that $\mathfrak {M},i \models v_i^j$; a contradiction. In the later case, consider the clause $\Box _F p_i \wedge \Box _P p_i \wedge v_i^j \rightarrow c_j$ (which was added by (C4)). Since $\mathfrak {M}, i \models \Box _F p_i \wedge \Box _P p_i$, we obtain that $\mathfrak {M},i \models c_j$; again a contradiction. This completes the proof of the claims (M1)–(M4).

It follows from (M1) and (M4) that for every *i* and *a* with $1 \le i\le n$ and $a \in \mathbb Z$ there is exactly one *c* with $1 \le c \le 3$, such that $\mathfrak {M},a \models v_i^c$. Moreover, because of (M2) the choice of *c* is independent of *a*. Accordingly, the coloring *f* that assigns the unique color *c* to every vertex $v_i$ such that $\mathfrak {M},a \models v_i^c$ forms a partition of the vertex set of *G*. Also *f* is a valid 3-coloring because for every $\{v_i,v_j\} \in E(G)$ it holds that $\mathfrak {M},a \not \models \lnot v_i^{c} \vee \lnot v_j^{c}$ for every $a \in \mathbb Z$ (using the clause added by C5) and hence $v_i$ and $v_j$ must be assigned distinct colors by *f*. $\square$

#### Corollary 11

Let $O\in \{{{\mathrm{\Box _{\mathrm {F}}}}},{{\mathrm{\Box _{\mathrm {P}}}}}\}$ then $\mathrm {Eval}^{O}(\textsc {krom})$ is $\mathsf {paraNP}$-complete (the $\mathsf {NP}$-completeness already holds for backdoor sets of size zero).

#### Proof

Satisfiability of  is $\mathsf {NP}$-hard [2, Theorem 5]. $\square$

## Conclusion and Discussion

We lift the well-known concept of backdoor sets from propositional logic up to the clausal fragment of linear temporal logic . From the investigated cases we obtain a comprehensive picture of the parameterized complexity for the problem of backdoor set evaluation. The evaluation parameterized by the size of the backdoor into $\textsc {krom}$ formulas becomes in all cases $\mathsf {paraNP}$-complete and as a result is unlikely to be solvable in $\mathsf {FPT}$ whereas the case of backdoor evaluation into the fragment $\textsc {horn}$ behaves differently. While allowing only  makes the problem fixed-parameter tractable, allowing both, ${{\mathrm{\Box _{\mathrm {F}}}}}$ and ${{\mathrm{\Box _{\mathrm {P}}}}}$, makes it $\mathsf {paraNP}$-complete. The last open case, i.e., the restriction to either ${{\mathrm{\Box _{\mathrm {F}}}}}$ or ${{\mathrm{\Box _{\mathrm {P}}}}}$ is open for further research and might yield an $\mathsf {FPT}$ result. We want to note here that all of our results still hold if LTL is evaluated over the natural numbers instead of the integers.

Satisfiability of  is $\mathsf {NP}$-complete, for $\textsc {horn}$/$\textsc {krom}$ it is in $\mathsf {P}$/$\mathsf {NL}$ [2]. With the help of our backdoor notion, we achieved for a $\textsc {horn}$-backdoor an $\mathsf {FPT}$ membership. However, for $\textsc {krom}$ this surprisingly was not possible ($\mathsf {paraNP}$-c., Theorem 9). For the “full global” fragment only for $\textsc {horn}$ satisfiability is in $\mathsf {P}$ and for $\textsc {krom}$ it is $\mathsf {NP}$-complete [2]. Here in both cases, our notion of backdoors was not fruitful. This is, however, natural since applying the backdoor approach to a novel problem is never a simple nor straightforward task. We see our work as a first attempt to come up with such a notion for , and, given the notorious difficulty of the -satisfiability problem, we believe our tractability result for  formulas restricted to the always operator that are almost $\textsc {horn}$ is an encouraging result that justifies further investigation of this approach. As mentioned earlier,  restricted to the always operator, is already pretty interesting, since it allows one to express “Safety” properties of a system (e.g., , where *x* encodes something bad to happen). Also, see the work of Kupferman and Vardi on this topic [24]. Moreover, our intractability results for the remaining fragments of  indicate that a different notion of “closeness” is required to obtain tractability results for these fragments.
